# Metal Complexes, an Untapped Source of Antibiotic Potential?

**DOI:** 10.3390/antibiotics9020090

**Published:** 2020-02-18

**Authors:** Angelo Frei

**Affiliations:** Centre for Superbug Solutions, Institute for Molecular Bioscience, The University of Queensland, St Lucia 4072, Australia; angelo.frei.ch@gmail.com

**Keywords:** antibiotics, antimicrobials, metal complexes, metal-based drugs, medicinal chemistry

## Abstract

With the widespread rise of antimicrobial resistance, most traditional sources for new drug compounds have been explored intensively for new classes of antibiotics. Meanwhile, metal complexes have long had only a niche presence in the medicinal chemistry landscape, despite some compounds, such as the anticancer drug cisplatin, having had a profound impact and still being used extensively in cancer treatments today. Indeed, metal complexes have been largely ignored for antibiotic development. This is surprising as metal compounds have access to unique modes of action and exist in a wider range of three-dimensional geometries than purely organic compounds. These properties make them interesting starting points for the development of new drugs. In this perspective article, the encouraging work that has been done on antimicrobial metal complexes, mainly over the last decade, is highlighted. Promising metal complexes, their activity profiles, and possible modes of action are discussed and issues that remain to be addressed are emphasized.

## 1. Introduction

At first sight it might seem that the antibiotic drug pipeline is slowly recovering after several decades of extreme drought. However, a closer look at the 42 compounds currently in clinical development highlights a major problem in the field. Only 11 of these compounds represent entirely new structural classes, while the rest are merely derivatives and modifications of already approved antibiotics [1]. While these derivatives represent a viable short-term solution, it is likely that bacteria will quickly develop resistance to these compounds as well. Another feature shared by all these molecules and most lead compounds in both preclinical and clinical development is that they are purely organic compounds. This observation can be rationalized by the fact that most of the chemistry we find in living organisms, from DNA to proteins and metabolites, is mostly based around carbon and a few other elements. At the same time, transition metals are generally thought to be toxic and only to be useful as catalysts or as materials in alloys, coatings, and electronic devices, etc. On the other hand, metals, while present in much smaller quantities, are just as essential for life. Without metals most enzymes would not be able to conduct their impressively fine-tuned transformations [2]. Metal compounds have access to modes of action that are difficult or even impossible to achieve with organic molecules alone. Furthermore, coordination compounds of metals have access to a vast variety of different geometries and generally possess a higher 3D character compared to the generally rather flat organic molecules. The three-dimensionality of molecules has repeatedly been associated with higher clinical success rates [3,4]. This is because the shape of a molecule is one of the crucial factors in determining its biological fate and activity [5,6,7]. Very recently, Morrison et al., reported a thorough analysis of the potential of metal complexes as ‘metallofragments’ for drug discovery. The authors found that metallofragments enable access to structural 3D space that is not covered by the vast majority of organic compounds [8].

Metal-containing compounds have played a small but seminal role in medicinal chemistry of throughout the 20th century. An arsenic-containing compound, Salvarsan, was discovered at the beginning of the century and became the first effective treatment of syphilis [9]. It was however the discovery of the anticancer drug cisplatin and its successors that really kickstarted the field of inorganic medicinal chemistry. Even today, platinum-based chemotherapeutics are still used in the majority of cancer treatments [10]. The gold-containing auranofin is an approved drug for the treatment of rheumatoid arthritis and is currently under investigation for its anticancer as well as antimicrobial properties [11,12,13,14,15,16]. Invigorated by these breakthrough successes, the field has expanded to many other elements in the last few decades, with complexes of titanium, iron, ruthenium, gallium, palladium, silver, gold, bismuth, and copper entering clinical trials [17,18,19,20,21,22]. The medicinal applications of these metal complexes range from anticancer to antimalaria over to neurodegenerative diseases. Strangely, antibacterial applications are remarkably sparse in this list and the number of literature reports on metal-based antimicrobials is dwarfed by the much more frequent publications on metal-based anticancer compounds. This is surprising as metals such as bismuth and silver have long been known to possess antibacterial properties. Some medicinal products are currently available. There are however a variety of products, such as silver-coated underwear, with a more dubious scientific basis. Nevertheless, the systematic evaluation of the antimicrobial properties of metal complexes has increased in pace over the last decade, with several reports highlighting the activity and potential modes of action of metal-based antibiotics. This perspective article will discuss the major discoveries in the non-traditional field of metal complex-based antibiotic compounds, focusing on the last decade and the most promising elements, with coverage of molecular metal-compounds but not supramolecular assemblies or nanoparticles which have been reviewed extensively elsewhere [23,24,25,26]. In general, the article will restrict itself to studies into metal complexes, i.e., compounds where the metal ion is more or less stably coordinated by one or multiple ligands. A recent review provides an overview of organometallic derivatives of known antibacterial drugs [27]. While some overlaps exist, this work focuses on all metal-containing small molecules and includes many important reports that were published in 2019. In conclusion, the advantages and disadvantages of metal-based drugs will be discussed and future promising directions for the development of the field in the coming years will be highlighted.

## 2. Silver

One of the first documented medicinal applications of silver was reported in 980 A. D. when Avicenna described the use of silver filings as a blood purifier for offensive breath and heart-palpitations [28]. In the 18th and 19th century, silver compounds found a range of applications such as the use of colloidal silver for wound antisepsis and silver nitrate for the treatment of burn wounds [29]. Even though the discovery of antibiotics diminished the use of silver, a range of silver-based compounds are still employed today for their medicinal properties [30]. Almost 300 clinical trials featuring silver-containing compounds and formulations for a range of different applications are currently ongoing or in the recruitment phase [17]. Silver sulfadiazine is approved by the Food and Drug Administration (FDA) in the US for its use as a broad spectrum topical antibiotic for some burn wounds [27,31]. However, some trials have suggested that silver-based medicines generally do not perform better than non-silver-based treatments. A 2017 report on a randomized control trial comparing polyhexanide/betaine gel with silver sulfadiazine in 46 adult patients with partial-thickness burns found no significant differences in healing times, infection rates, and treatment costs in both groups. However, the pain score of the polyhexanide/betaine gel group was shown to be lower than in the silver sulfadiazine group [32]. In a two-arm open label multicenter randomized controlled trial in 2019, 89 adult patients with acute partial thickness burns were treated either with Flaminal^®^ Forte (hydrated alginate polymers with a biologic enzyme system based on glucose oxidase and lactoperoxidase stabilized by guaiacol) or Flamazine^®^ (silver sulfadiazine). No significant difference was found in the average wound-healing time, but Flaminal^®^ Forte was deemed advantageous due to less frequently required dressing changes [33]. Finally, a 2019 meta-analysis of 81 studies found that 11/11 studies that met the inclusion criteria of the review found silver sulfadiazine to be inferior to alternative treatments in the mean wound-healing time [34].

In the last 20 years, some new classes of silver complexes have garnered attention for their antibacterial properties, in particular *N-*heterocyclic carbene (NHC) complexes of silver(I). A comprehensive summary of antimicrobial silver compounds can be found in several recent reviews [27,35,36,37]. While some complexes from the late 1990s and early 2000s showed promising activity against a range of bacterial strains, no significant developments have been reported in the last years, possibly due to the fact that the exact mode of action of silver compounds remained largely unknown. It was suspected that the observed antibacterial effect is caused by the Ag(I) ions being released through dissociative mechanisms after entering the bacteria as coordination complexes. In 2019 the group of Sun published several ground-breaking studies on the molecular bacterial targets of a series of metals, including silver. By integrating gel electrophoresis with inductively coupled plasma mass spectrometry (GE-ICP-MS), the group identified 34 unique proteins targeted by silver ions (originating from AgNO_3_) in *Escherichia coli,* many of which play a role in glycolysis and the tricarboxylic acid (TCA) cycle [38]. Amongst these proteins, glyceraldehyde-3-phosphate dehydrogenase (GAPDH) was studied in-depth due to its importance in glycolysis. Extensive biochemical analysis combined with X-ray crystal structures of Ag(I) ions coordinated to an active site cysteine demonstrated the inhibitory role of silver ions, leading to the first identification of a bona fide molecular target of silver in bacteria. Since GAPDH binding sites are conserved among humans and bacteria, delivery systems will have to be tailored towards the latter for silver ions to be further developed as antibiotics [39]. As many of the identified targets are part of the Krebs cycle, the team hypothesized and demonstrated that supplementing the silver nitrate treatment of *E. coli* with metabolites involved in the early stages of the Krebs cycle significantly increased the antimicrobial effect of the silver compound. In most studied silver complexes, the observed antibacterial activity is ascribed to silver ions being released inside of the bacteria, suggesting that the targets identified by the group of Sun are likely also valid for metal complexes and not just silver salts such as AgNO_3_ [27,35−37]. Future work on silver complexes will have to focus on improving selective uptake and accumulation of silver through rational ligand design.

The studies by the group of Sun exemplify the type of work that needs to be done for metal-based compounds to be considered seriously as potential antibiotics. More work in this vein will hopefully follow in the future.

## 3. Gold

**Figure 1:**
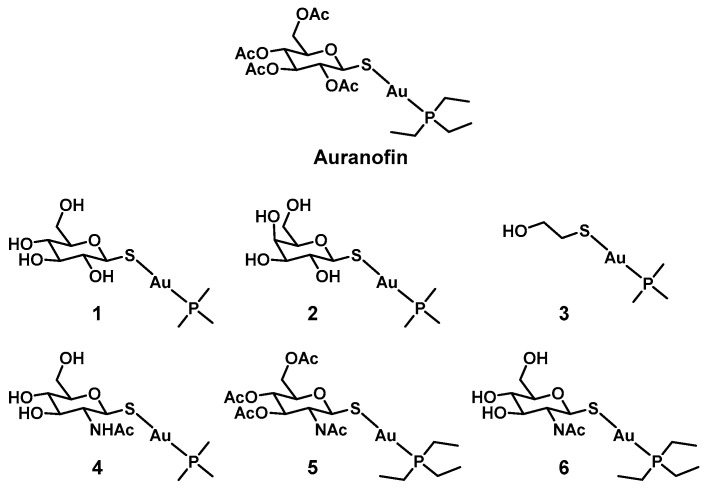
Structures of auranofin and its derivatives.

Similarly to silver, gold has garnered attention for its medicinal properties early on. Robert Koch described the activity of potassium dicyanidoaurate(I) (K[Au(CN)_2_]) against *Mycobacterium tuberculosis* in 1890 [40]. The antimicrobial properties of gold complexes have been summarized in recent review articles [41,42,43]. NHC complexes of gold in particular seem to possess promising antibacterial activity. However, there is a general lack of more in-depth studies beyond simple minimum inhibitory concentration (MIC) measurements in this area. In most cases where more experiments where conducted, these compounds were found to possess significant cytotoxicity against eukaryotic cells, suggesting a nonspecific mode of action rendering them unsuitable for antibacterial applications. One notable exception to this trend is auranofin (Figure 1). Auranofin is a gold-based FDA-approved antirheumatic drug. It is also currently in clinical trials for its potential anticancer applications [17]. Recently it was found to also be effective against a series of clinically relevant drug-resistant Gram positive (Gram(+)) species (*Staphylococcus aureus*, methicillin-resistant *S. aureus* (MRSA), *Enterococcus faecium*, and *Enterococcus faecalis*), as well as *M. tuberculosis* [15]. In contrast, almost no activity was found against Gram negative (Gram(−)) species (MIC ≥ 16 µg/mL against *Acinobacter baumannii*, *Pseudomonas aeruginosa,* and *Klebsiella pneumoniae*). A known cellular target of auranofin is thioredoxin reductase (Trx), an essential source of reducing equivalents in Gram(+) bacteria and *M. tuberculosis*. It is proposed that the glutathione system present in Gram(−) bacteria can compensate for the loss of the reducing ability of Trx. Indeed, assays using a glutathione knockout (*ΔgshA*) mutant strain of *E. coli* resulted in higher auranofin activity. A combination of auranofin with paraquat (which generates reactive oxygen species, ROS) against *S. aureus* showed significant synergetic activity (~5-log decrease in colony forming units, CFU) whereas paraquat alone had only minor antimicrobial activity (<1 log decrease in CFU). In this case it seems that the combination of increased ROS and compromised cellular defenses against oxidative stress resulted in increased bacterial death [15]. Other studies hypothesized that the outer membranes of Gram(−) species are able to prevent auranofin accumulation. This is supported by the finding that co-administration of auranofin with membrane permeabilizing antibiotics such as polymyxin B lead to restored activity against Gram(−) bacteria [44,45].

It was further shown that auranofin was effective at treating MRSA in a murine systemic infection model. The animals were given a single i.p. injection at either 0.12 or 0.012 mg/kg once a day (the maximum tolerated dose in mice was 70 mg/kg). Four out of eight and three out of eight mice of the respective doses survived to day 7, whereas none of the animals in the vehicle control group survived beyond day 4. Although auranofin displays a low therapeutic index toward eukaryotic cells in vitro, chronic auranofin exposure was found to be safe for patients while taken over extended periods of time, with no cumulative toxicity observed over 5 years [46]. More recent work by Tharmalingam et al., found that no resistance could be detected in a *S. aureus s*train after 25 days of auranofin exposure [47]. Another report by the group of Wu describes potent activity of auranofin against biofilms of *S. aureus* and *E. faecalis* as well a synergistic microbicidal effect with linezolid, fosfomycin, and chloramphenicol in vitro and in vivo [48]. Auranofin has the added advantage of being already approved by the FDA, which could accelerate the expansion of its use to bacterial infections [15]. From these initial promising results, some groups have embarked on a quest to improve the properties of auranofin, with emphasis on reducing mammalian cytotoxicity. The group of Yan prepared 40 auranofin analogues to determine their Gram(+), Gram(−), and cytotoxic activity, establishing a structure–activity relationship. Compounds (**1–4**) possessed a broader activity spectrum, with bactericidal activity against *S. aureus*, *A. baumannii, Enterobacter cloacae*, *E faecium*, and *E. coli*, with associated half maximal inhibitory concentration (IC_50_) values between 50 and 100 µM against A549 cells (Table 1) [16]. For the Gram(+) bacteria tested, the cytotoxicity was around two orders of magnitude lower than the antibacterial activity, indicating a favorable therapeutic index. The activity could be improved against Gram(−) species, however, the therapeutic index was significantly smaller. Some of the compounds in this report (**4–6**) were further shown to be effective inhibitors of the gastric pathogen *Helicobacter pylori* in a concurrent publication [49]. Overall, these findings suggest that the properties of auranofin can be optimized further for antibacterial applications. While gold is a rather expensive metal, the fact that auranofin is already FDA-approved could significantly facilitate the road to approval for gold-based antibiotic compounds.

## 4. Gallium

**Figure 2:**
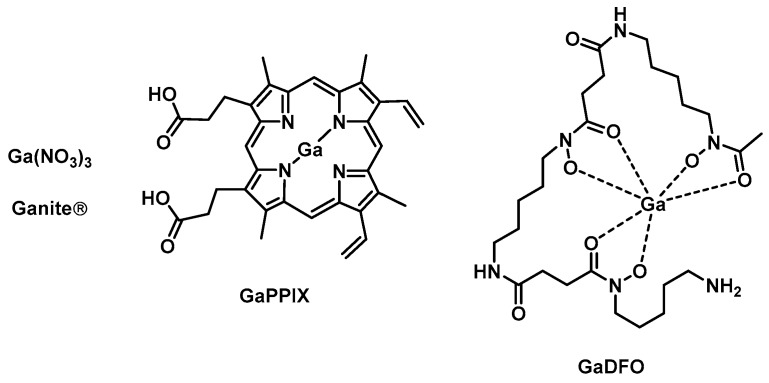
Examples of gallium compounds with antibacterial activities.

The gallium formulation **Ganite** is an FDA-approved bone resorption inhibitor based on Ga(NO_3_)_3_ that was recently shown to possess promising activity against several bacterial species such as *P. aeruginosa*, *A. baumannii*, and *M. tuberculosis* [50]. Generally, gallium compounds target the iron metabolism of bacteria due to their similarity to iron. When Ga(III) is incorporated into iron-dependent enzymes, it cannot be reduced to Ga(II), effectively inhibiting the enzyme. Gallium protoporphyrin IX (**GaPPIX**, Figure 2) is thought to inhibit the iron metabolism by targeting heme uptake and has been shown to have good activity against both Gram(+) (various methicillin-sensitive *S. aureus* (MSSA) and MRSA strains, MICs *=* 0.031–0.062 µg/mL) and Gram(−) bacteria (*P. aeruginosa*, *K. pneumoniae, A. baumannii,* MICs *=* 4–16 µg/mL) [50]. On the other hand, sources of free gallium such as Ga(NO_3_**)_3_** are taken up by siderophore-mediated and/or free iron uptake pathways. A combination of the two types of gallium to achieve maximum effect is therefore desirable. Application of both **GaPPIX** and Ga(NO)_3_ resulted in synergetic activity against MRSA and significantly reduced bacterial populations in *K. pneumoniae* and *A. baumannii* biofilms [51]. More recently, a topical chitogel-deferiprone **GaPPIX** treatment was shown to reduce bacterial biomass in *S. aureus* biofilms in a sheep sinusitis model [52].

Gallium desferrioxamine (**GaDFO,** Figure 2) was designed to target the iron DFO uptake pathways present in *P. aeruginosa* [53]. In 2008, Banin et al., reported that **GaDFO** was able to efficiently kill both planktonic *P. aeruginosa* as well as mature *P aeruginosa* biofilms. A strong synergetic effect was observed with the antibiotic gentamicin. In vivo studies in a rabbit kerititis model showed that the combination of **GaDFO** with gentamicin was able to reduce the bacterial infiltration and final scar area by 50%–60%. However, it remained unclear whether this reduction was solely attributable to the **GaDFO** [54].

It has to be noted that most studies of the antimicrobial activity of gallium compounds employ iron-poor media, usually via addition of an iron chelator, as high concentrations of iron have been shown to reduce gallium activity. Other work found that the activity is improved further when the studies are conducted in human serum, however exogenous iron addition could nullify gallium compound activity in these cases [50,55]. A comparative study of Ga(NO_3_)_3_, **GaPPIX** as well as a third gallium-based agent, Ga(III)-maltolate (GaM), investigated the antimicrobial activity of these three compounds against ESKAPE (*E. faecium*, *S. aureus*, *K. pneumoniae*, *A. baumannii*, *P. aeruginosa*, and *Enterobacter***)** pathogens under different growth conditions. Importantly, it was shown that the more ‘labile’ compounds Ga(NO_3_)_3_ and GaM lost their antibacterial activity completely in Mueller Hinton broth (MHB) and iron-depleted MHB. **GaPPIX** showed some bactericidal activity under these conditions against several *S. aureus* (MICs = 0.06–0.12 µM) and *A. baumannii* (MICs = 16–32 µM) strains. On the other hand, bacteriostatic activity of Ga(NO_3_)_3_ and GaM was found against some strains (mainly *A. baumannii* and *P. aeruginosa*) in RPMI-1640 supplemented with 10% human serum which better simulates the low iron content and presence of human serum in in vivo environments. Conversely, **GaPPIX** lost all activity against *S. aureus* (MIC > 128 µM) and showed more a more variable profile against *A. baumannii* (MICs = 0.25–128 µM) [56].

Preliminary in vivo studies by Goss et al., showed that gallium nitrate displayed antibiotic activity in murine lung infections and improved lung function in a preliminary phase I clinical trial, although no placebo control group was used in this study. The group also found that *P. aeruginosa* developed resistance to gallium with similar frequency as to other approved antibiotics [57]. A phase II clinical trial (IGNITE) of gallium nitrate in adults with cystic fibrosis found that there was no significant difference between the number of responders (defined as a participant having a 5% or greater increase in lung function by day 28) in the intravenous (IV) gallium nitrate and placebo group. However, the study did find that a significantly greater reduction of *P. aeruginosa* was found in the sputum of gallium treated patients compared to the placebo group [58]. Furthermore, gallium nitrate was found to be safe and well tolerated by patients [57,58].

Recently, Pandey et al., described the preparation of a theranostic gallium siderophore ciprofloxacin conjugate. This compound was found to possess good antibacterial activity against both Gram(+) and Gram(−) strains (*E. coli* K12, S*. aureus* RN4220, *P. aeruginosa* PA01, and *K. pneunomiae* CRE-11; MICs = 0.23–12.5 µM) in iron-deficient media. By using radioactive ^67^Ga, the group was able to follow the metabolic fate of the compound in mice, opening up the door for potential theranostic applications, with both diagnostic and therapeutic benefits in bacterial infections [59].

Despite these promising results, there is a general lack of knowledge around the exact molecular mechanisms of action of gallium-based compounds. As mentioned earlier, Ga(III) has very similar properties to Fe(III) and can disrupt a variety of iron-dependent functions as it cannot be reduced under physiological conditions [60]. Recent studies by the group of Hongzhe Sun elucidated the molecular targets of gallium. By integrating metalloproteomics with metalbolomics and transcriptomics, the group identified *Pa*RpoB and *Pa*RpoC as binding sites for gallium nitrate in *P. aeruginosa*. The targets were validated by overexpressing the respective genes in *E. coli*. Only cells with *Pa*RpoB and *Pa*RpoC could be fluorescently labelled by the group’s Ga(III) probe. These proteins are two subunits of the DNA-dependent RNA polymerase, an essential enzyme in transcription and gene expression. Through cellular thermal shift assays, these proteins were found to be destabilized upon binding to gallium. By adding different concentrations of exogenous metabolites, the group found that acetate in combination with gallium seemed to increase its antibacterial potency. Indeed, this combination treatment increased the uptake of gallium into the bacteria significantly. This effect could also be replicated in cell and murine infection models, indicating a potentially promising combination therapy for bacterial infections [61].

Analogous to silver, it seems that the antibacterial effect of gallium compounds stems from the binding of the free Ga(III) ion to specific targets in bacteria. Preparation of stable or semi-stable gallium coordination complexes could improve the availability and uptake of gallium. These ligands should still allow for a dissociation of the gallium core once the compound has entered the bacteria. To increase bacterial specificity, ligands which allow for derivatization (e.g., with targeting units) are therefore desirable.

## 5. Bismuth

**Figure 3:**
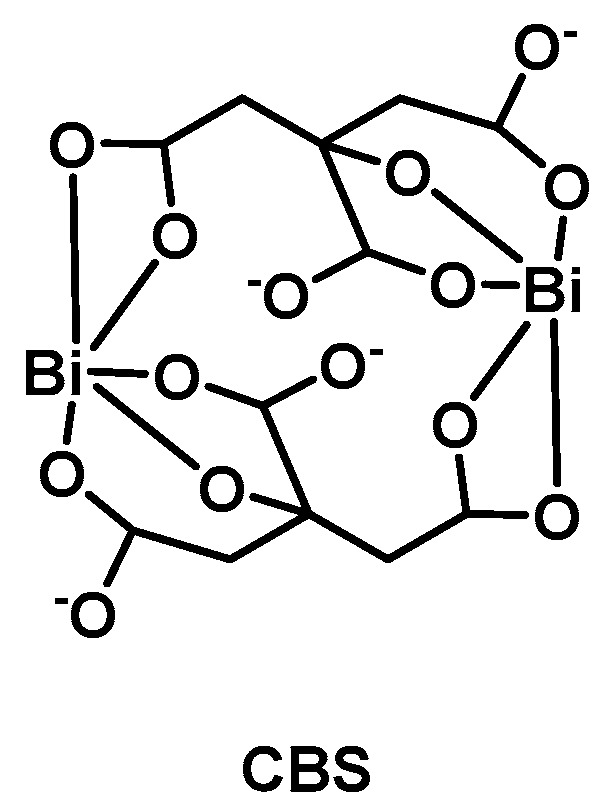
Structure of colloidal bismuth subcitrate (**CBS**).

Another metal that has long been known to possess beneficial medicinal properties is bismuth. Similarly to silver and gallium, however, the molecular mechanism of bismuth-based compounds has remained elusive for a long time. Bi(III) exhibits remarkably low toxicity against humans while being potently toxic against bacteria. Bismuth and its complexes have reportedly been used in the treatment of syphilis, colitis wound infection and quartan malaria. Its most prominent use to date however is for gastrointestinal disorders [62]. *H. pylori* has been shown to be particularly susceptible to bismuth, with three drugs, bismuth subsalicylate (Pepto-Bismol), colloidal bismuth subcitrate (**CBS**, De-Nol, Figure 3), and ranitidine bismuth citrate (Pylorid), used to treat their infections [63,64]. Bismuth compounds with antimicrobial and anticancer activities have been reviewed recently [65]. The group of Sun and co-workers uncovered that glutathione binds Bi(III) in human cells and compartmentalizes it into subcellular vesicles, effectively removing it from intra- and extracellular compartments. [66] Over the years, the use of GE-ICP-MS techniques and customized fluorescent probes has facilitated the identification of a number of Bi-binding proteins. Evidence points to multiple modes of action with several targets, which is in agreement with the low resistance frequency found with bismuth compounds [67]. In 2018, the group of Sun described a repurposed application of the bismuth drug CBS. The compound was shown to irreversibly inhibit the metallo-β-lactamases (MBL) such as New Delhi MBLs (NDMs), Verona integron-encoded MBLs (VIMs), and imipenemases (IMPs). Crystallography studies revealed that Bi(III) can replace one of the Zn(II) ions in the active site of MBLs. Furthermore, cotreatment of CBS with meropenem restored efficacy in vitro and in vivo and significantly slowed down further resistance development in NDM-1 positive *E. coli* bacteria [68]. Since CBS is already a clinically approved drug and has proven safe in humans at high doses, its path to approval as a MBL inhibitor may be facilitated. At the same time the wealth of recent molecular insights into the modes of action of bismuth will allow for the development of more rationally designed compounds that may display even better activity profiles.

## 6. Ruthenium

**Figure 4:**
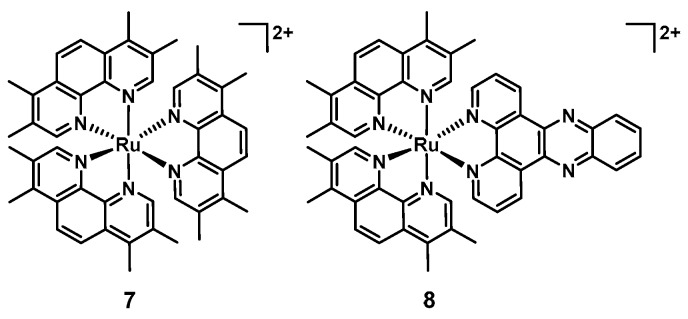
Structures of [Ru(Me_4_phen)_3_]^2+^ (**7**) and [Ru(2,9-Me_2_phen)_2_(dppz)]^2+^ (**8**).

Ruthenium complexes have been widely studied for their biological activity, particularly their anticancer properties. Labile ruthenium complexes have been shown to bind nucleic acids coordinatively through ligand exchange reactions. On the other hand, inert compounds, generally bearing one or more polypyridyl ligand(s) can bind DNA and RNA through intercalation [20,21]. The biological properties of ruthenium, including some interesting antimicrobial properties were already described in the 1950s [69]. Amongst several transition metals tested, it was shown that [Ru(Me_4_phen)_3_]^2+^ (**7**, Figure 4) showed remarkable antimicrobial activity in vitro. However, it was also noted that the compounds were not effective in vivo due to rapid clearance following IV administration in mice, leading the authors to suggest topical applications. Interestingly only minimal increases in MIC were detected after 25 passages with the ruthenium complex **7**, compared to over a 10′000-fold increase in MIC with penicillin against *Streptococcus pyogenes*, highlighting potential benefits of ruthenium-based antibiotics with regards to their propensity to induce resistance [70,71,72].

In 2011 Aldrich-Wright and co-workers reported that [Ru(2,9-Me_2_phen)_2_(dppz)]^2+^ (**8**, Figure 4) possessed activity against a range of Gram(+) bacteria (MSSA, MRSA, and *Bacillus subtilis* MICs = 2–8 µg/mL) in vitro and in vivo, increasing the survival of a *S. aureus-*infected *Caenorhabditis elegans* population when supplied at doses of 8 µg/mL or higher. These compounds have been shown to be able to bind DNA through intercalation, however it remains unclear whether this is their effective mode of action. Importantly, the tested compound was not toxic to *C. elegans*. On the other hand, no activity was observed against *E. coli*. [73].

Ruthenium polypyridyl complexes can also act as photosensitizers, generating ROS upon light irradiation, an approach which is known as antibacterial photodynamic therapy (aPDT) in the realm of infectious disease treatments [74]. ROS can damage enzymes, proteins, DNA and/or RNA if they are generated in their close proximity. The propensity of these complexes to bind DNA/RNA makes these likely targets for this class of ruthenium compounds. The potential for aPDT is particularly promising for localized infections, where conventionally a systemic antibiotic would be administered [75]. Traditional targets include skin and wound infections but thanks to advances in endoscopes and fiber optic devices, most body areas e.g., ear, nose oral cavity, gastrointestinal tract, urogenital tract, and lungs are now accessible to localized light irradiation for aPDT [76]. Furthermore, early studies into the resistance induction of aPDT have found little to no resistance development in treated bacterial strains [77].

**Figure 5:**
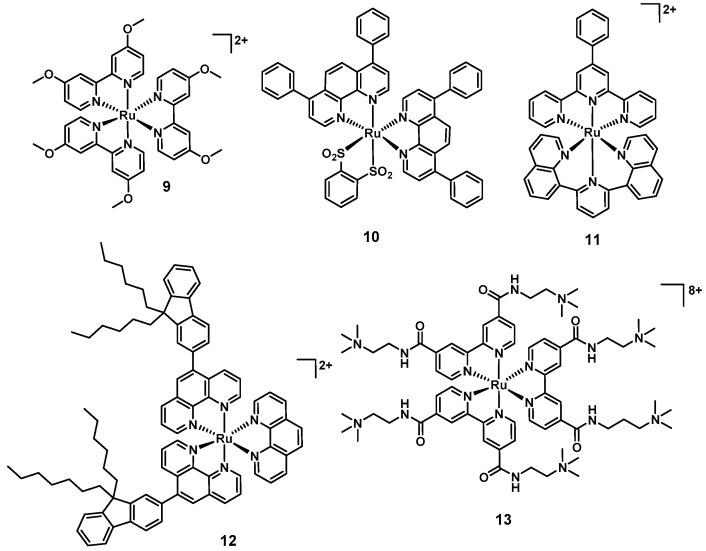
Structures of aPDT-active ruthenium polypyridyl complexes.

Donnely et al., were amongst the first to report the aPDT potential of a ruthenium polypyridyl (**9**, Figure 5) complex in 2007. The compounds showed MIC values of 12.5 µg/mL, 50 µg/mL, and ≤12.5 µg/mL against *S. aureus*, *P. aeruginosa,* and *C. albicans,* respectively, upon white light irradiation. Unfortunately, no toxicity studies against human cell lines were reported [78]. The group of Gasser reported complexes with light-mediated activity against bacteria in 2014. Compound **10** showed good activity against *S. aureus* (>6 log reduction at 50 µM), while **11** was active against both *S. aureus* and *E. coli* when irradiated with 420 nm light (>6 log reduction at 50 µM; Figure 5) [79]. No activity was observed in the absence of light. However, **10** showed activity against both normal lung fibroblasts (MRC-5, IC_50_ = 15.6 µM) and human cervical carcinoma cells (HeLa, IC_50_ = 5.7 µM) after 48 h of incubation in the dark (**11** was nontoxic up to 100 µM in the absence of light). More recently, Le Gall et al., reported a structure–activity relationship of 17 different light-activated ruthenium complexes (**12** is shown as an illustrative example, Figure 5) with a range of activity profiles against various Gram(+) and Gram(−) strains (**12** led to a 5 log reduction in growth in both *S. aureus* RN4220 and MRSA N315) [80]. In 2019 Feng et al., described a series of charged ruthenium complexes that showed good activity against *S. aureus* and MRSA upon light irradiation (only minor activity against Gram(−) *E. coli* was found). The most highly charged complex **13** was shown to possess the best antibacterial activity, displaying 6–7 log reduction in bacterial viability (comparable to methicillin and vancomycin at equal concentrations). Scanning electron microscopy (SEM) experiments revealed damaged and deformed cell walls in *S. aureus,* pointing to the highly negatively charged bacterial surface as the target of this class of compounds. Interestingly, co-culture experiments revealed preferential killing of bacterial cells over mammalian cells in vitro [81].

Overall, most reported ruthenium aPDT agents (except **10**) possess an overall positive charge, which may promote interactions with the negatively charged bacterial membrane.

**Figure 6:**
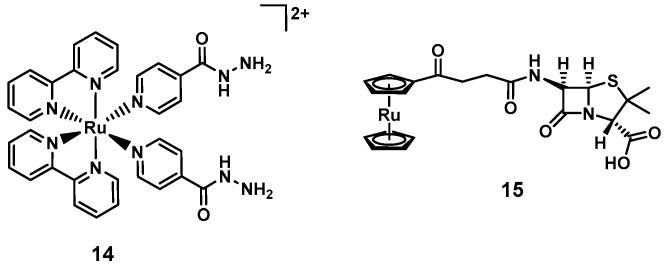
Ruthenium polypyridyl complex with light-triggered isoniazid ligand release (**14**) and a ruthenocene derivative of a β-lactam (**15**).

Smith and Zhang et al., pursued a different light-mediated strategy, preparing a ruthenium complex (**14**, Figure 6) where a ligand, namely the anti-tuberculosis drug isoniazid, is released upon light irradiation 465 nm. This compound was found to be inactive against *E. coli* and *B. subtilis* but highly selective towards *Mycobacterium smegmatis*, where a survival of <1% was observed at 10 µM upon light irradiation (MIC*_M. smegmatis_* = 4 µM, MIC(isoniazid)*_M. smegmatis_* = 29 µM). At the same time, the compound was found to be non-toxic to mammalian cells (>90% survival of MRC-5 cells at 200 µM) [82].

In a different approach, ruthenocene was conjugated to the β-lactam 6-aminopenicilinic acid. The resulting complex (**15**, Figure 6) showed antibacterial activity against a range of MSSA clinical isolates, *S. epidermidis,* and *E. faecalis*, with MIC values ranging from 0.5 to 16 µg/mL. No activity was observed against MRSA (MIC = 256 µg/mL) [83]. Interestingly the corresponding ferrocene analogue displayed lower activity, potentially due to its higher susceptibility to oxidation [84]. The authors were also able to obtain a co-crystal structure of **9** with CTX-M β-lactamase at 1.18 Å resolution. This highlights another advantage that metal-complexes offer: Their increased propensity for crystallization with higher associated electron density of the metal center facilitates the resolution of protein target structures.

**Figure 7:**
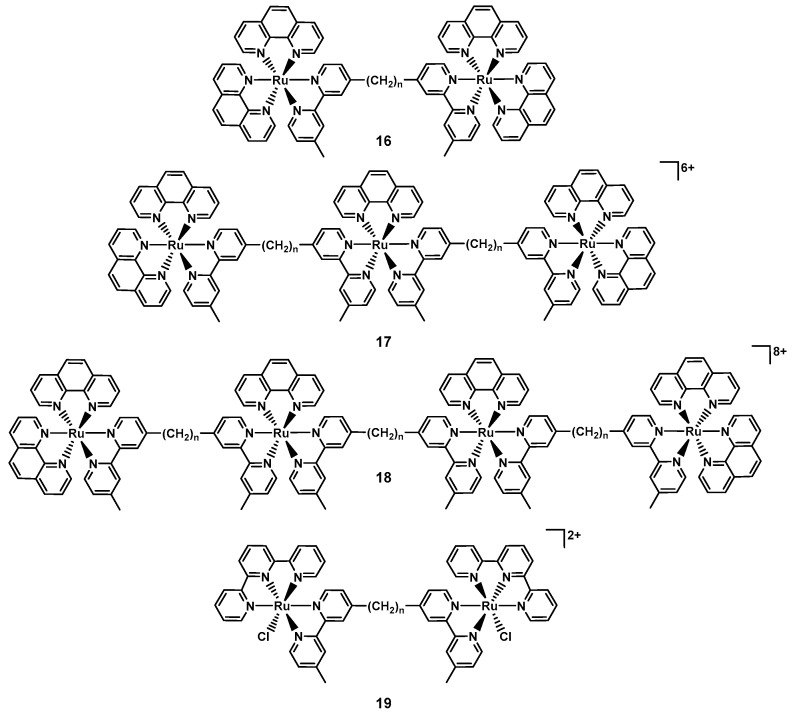
Example structures of the polynuclear ruthenium complexes reported by the groups of Keene and Collins (*n* indicates the length of the alkyl chain).

The first investigations into the antibacterial properties of dinuclear ruthenium complexes of the type [Ru_2_(phen)_4_(µ-bb_n_)_2_]^4+^ (bb_n_ = bis [4(4′-methyl-2,2′-bipyridyl)]-1,*n*-alkane, **16_n_**, Figure 7) were published by the groups of Collins and Keene in 2011. The group was able to isolate both the ΔΔ and ΛΛ enantiomers, observing slight differences in their antibacterial profile. The compounds were found to be active against both Gram(+) and Gram(−) bacteria as soon as the alkyl chain reached a certain length (e.g., for **ΛΛ16_7_**: MIC*_S. aureus_* = 64 µg/mL, and for **ΛΛ16_10_**: MIC*_S. aureus_* = 8 µg/mL), with somewhat lower activity against Gram(−) bacteria. Overall, the compounds showed significantly lower toxicity towards human acute monocytic leukemia cells (THP-1, model for nucleated eukaryotic cells) [85]. In following work, the authors found that longer alkyl chain length correlated positively with higher cellular uptake. As the longer chain length leads to more lipophilic compounds, the authors concluded that this increase in lipophilicity is responsible for the higher uptake [86]. Later studies showed that **10_16_** preferentially binds RNA in live bacteria and accumulates at the ribosomes, condensing them when they form polysomes. This finding suggests that the compounds halt translation of RNA and thereby protein synthesis in bacteria [87]. The group then went on to investigate the corresponding tri- and tetra-nuclear ruthenium compounds (**17_n_** and **18_n_**, Figure 7), showing that these complexes showed up to four-fold better activity compared to the dinuclear ones. Extensive NMR studies and molecular dynamics simulations revealed that complex **17_12_** could insert into a negatively charged phospholipid bilayer mimic of a bacterial membrane, suggesting membrane disruption as a possible mode of action of this class of compounds. Interestingly, no insertion was observed with eukaryotic membranes [88]. Generally, slightly lower activity was observed in Gram(−) strains compared to Gram(+) ones, even though the cellular accumulation was found to be similar [89]. Amongst these, *P. aeruginosa* was found to be notably less sensitive to these inert polynuclear ruthenium complexes despite similar cellular accumulation, indicating some inherent resistance to these compounds by this strain [72,90].

The same group also reported on dinuclear ruthenium complexes of the type [Ru_2_(tpy)_2_(µ-bb_n_)Cl_2_]^2+^ (tpy = 2,2′:6′,2”-terpyridine, **19**, Figure 7), where the labile chlorido ligand is aquated in solution generating a more highly charged complex. The compounds with a linker chain length of 7, 12 or 14 showed good antibacterial activity with MIC values between 1 and 8 µg/mL against MSSA, MRSA, and *E. coli*. Overall, the authors reported slightly reduced uptake and a concurrent small reduction in activity compared to the inert analogous complexes. This suggests that there is a fine balance between charge, charge separation and lipophility that ultimately affects both cellular uptake and antibacterial activity [91]. It also indicates that we do not understand the structure activity relationships of these compounds well enough yet to make predictions about what factors are decisive in a metal complex’s antibiotic activity.

**Figure 8:**
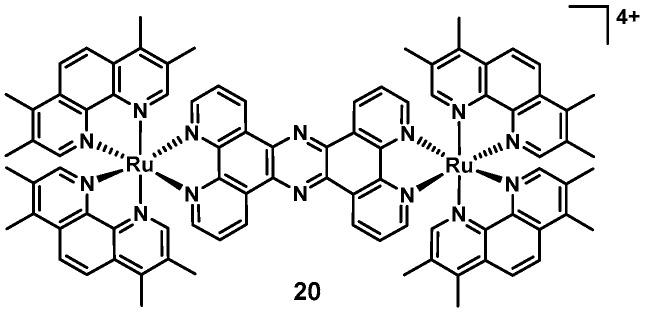
The dinuclear, tetracationic ruthenium polypyridyl complex reported by Smitten et al. [94].

In 2019, Smitten et al., reported the remarkable antimicrobial activity of a dinuclear ruthenium complex (**20**, Figure 8). This complex showed good antibacterial activity against pathogenic, multidrug resistant Gram(−) bacteria (*E. coli and E. faecalis*, MICs = 0.5–1.6 µM), while showing no significant cytotoxicity against eukaryotic cells (IC_50_ = 135 µM, HEK293). The authors showed that the complex could disrupt the Gram(−) membranes, as evidenced by changes in cell morphology and lump formation after 20 min. The compound seemed to accumulate at the cell poles, similarly to what had been reported for the polynuclear ruthenium complexes by Keene and Collins [92]. In follow-up work, **20** was shown to possess increased in vitro activity against *S. aureus* (SH1000) in chemically defined minimal media (CDM, MIC = 4 µM) compared to the more commonly used Mueller–Hinton-II media (MH-II, MIC = 40 µM), possibly due to more interactions with media substrates in the latter. STED nanoscopy, membrane damage assays and transmission electron microscopy studies suggest that the compound targeted both the bacterial membrane as well as the DNA-content of the cells in *S. aureus*. Furthermore it was shown that in contrast to the results with Gram(−) strains, **20** showed decreased activity against MRSA and an antimicrobial resistant (AMR) clinical isolate. The authors went on to conduct experiments with mutated *S. aureus* strains. The *tarO* strain is deficient in wall teichoic acids and the *dltA* strain is specifically deficient in *D*-alanylated teichoic acids. The MICs against both mutants were lower than against the original SH1000 strain, suggesting that **20** could potentially be binding to teichoic acids within the Gram(+) cell wall, reducing its ability to penetrate the bacteria and lowering its potency. Up-regulation of *mprF* has been found to be a frequent resistance mechanism in *S. aureus* against membrane active agents [93]. This upregulation results in a higher concentration of positive charges on the outer cytoplasmic membrane of *S. aureus* and reduces susceptibility to cationic compounds. The MIC against the *S. aureus* *ΔmprF* strain was significantly lower (1.5 µM) than in the SH1000 strain (40 µM) [94]. Altogether this study showed that the lower susceptibility of the drug-resistant Gram(+) *S. aureus* to **20** compared to the Gram(−) strains can be attributed to their different molecular membrane structures of these bacteria. These insights will be helpful in the design of the next generation of ruthenium-based antibacterial agents.

Overall the prominent role of ruthenium in anticancer applications has somewhat carried over to antimicrobial applications, making it one of the more intensely investigated elements in this field. The more detailed mode of action studies that have appeared in recent years provide promising groundwork towards understanding how these complexes work and designing the next generation of compounds. For ruthenium complexes to be developed further, in vivo efficacy experiments will be imperative, as there are only very limited data available at this stage and future research will have to focus on this area.

## 7. Iridium

**Figure 9:**
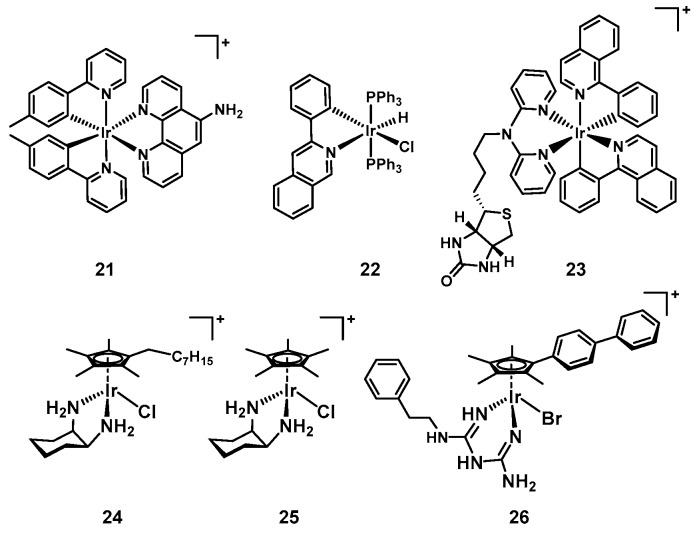
Selected iridium-based antibacterial complexes.

In the aforementioned reports on chlorido-substituted dinuclear ruthenium polypyridyl complexes, the authors also prepared the analogous iridium compounds. Interestingly the iridium compounds showed some degree of inhibitory activity but further studies revealed that the complexes were bacteriostatic as compared to the bactericidal ruthenium counterparts [91]. The origin of the difference in activities between the two metals is unknown at this stage. The Ir(III) compounds possess an overall charge of +4 before aquation of the chloride ligand (compared to +2 for the Ru(II) complexes), which may affect their ability to penetrate the bacteria. Furthermore, the chlorides of the iridium complexes were found to be more labile than the ruthenium one, suggesting that these compounds actually possess a +6 charge in solution, which might prevent high enough accumulation for bactericidal activity. In subsequent work, analogous iridium compounds generally performed worse than the ruthenium complexes, showing either lower or no antibacterial activity at all [95]. In 2015, Lu et al., reported on cyclometallated polypyridyl iridium complexes (**21**, Figure 9) that showed promising activity against *S. aureus*, but none against *E. coli*, *E. faecalis*, and *K. pneuomonia*. However, the most antibacterial compound (**21**, MIC*_S. aureus_* = 3.6 µM) was similarly cytotoxic against cancer cells, suggesting a non-specific toxic mode of action [96]. The same year, Jain et al., described another series of iridium(III) complexes with **22** showing promising activity against *E. coli* and *B. subtilis* (MIC*_B. subtilis_* = 4 µg/mL, MIC*_E. coli_* = 4 µg/mL) and demonstrated the ability to intercalate DNA [97]. A different cyclometallated iridium(III) dipyridylamine complex conjugated to biotin (**23**) was shown to effectively kill *P. aeruginosa*, a notoriously hard to kill Gram(−) species (MIC = 4 µg/mL). Two other analogues with no conjugated unit or a glycoside attached showed no activity against these bacteria. Upon irradiation with blue light, these two complexes decreased the survival of *P. aeruginosa* down to 2 ± 1% (methyl substituted) and 6 ± 2% (glycoside substituted) while the biotin conjugate showed no significant decrease in bacterial survival [98]. Some cyclometallated Ir(III) complexes are known to act as photosensitizers, generating ROS upon light irradiation, making them potential candidates for aPDT as well [99]. This work seems to indicate that biotin conjugation may be another possibility to improve bacterial uptake of metal complexes to further improve their activity.

The groups of Falkinham and Merola have previously investigated piano-stool type complexes of iridium and rhodium with amino acid ligands for their antimycobacterial activity, reporting better activity for the complexes bearing hydrophobic amino acids. The presence of a cyclopentadiene (Cp) ligand was also found to correlate with better antimycobacterial activity [100]. Follow up studies reported a series of pentaalkylcyclopentadienyl iridium and two cobalt complexes with three iridium and one cobalt compound possessing promising activity against nine *S. aureus* strains, including seven MRSA strains derived from patients and laboratories (**24**, MICs = 4–8 µg/mL, Figure 9). The active iridium complexes did not show any cytotoxicity against the Vero cell line ATCC CCL-81 up to 500 µg/mL or hemolytic properties up to 250 µg/mL. Complex **24** was further described as non-toxic in mice at doses of 5 mg/kg, however no experimental details were disclosed [101]. More recently, the same authors reported a new series of piano-stool iridium diamino-type complexes that showed activity against a *S. aureus* as well as a MRSA strain while displaying no toxicity in vitro and in vivo (**25**, MICs = 5–7.5 ug/mL, Figure 9). It was shown that the free diaminocyclohexane ligand did not possess any activity, hence the observed effect was attributed to the complex itself. Their exemplary study demonstrating the safety of a metal-based antibiotic in mice represents one of the first of their kind for iridium antibacterial agents. The complex did not cause any acute effects when given as a single IV dose at 2.5 and 5 mg/kg to adult outbred white mice (ICR strain), and the animals displayed normal growth, health, and behavior in the following 14 days. Through ICP-OES it was shown that the iridium compounds were excreted efficiently through their urine and no iridium was detectable following sacrifice of the mice on day 14. No data on in vivo efficacy was reported in this study [102].

Finally, the group of Sadler reported a series of 14 organoiridium(III) complexes in 2018. Compounds in this series displayed excellent activity against MRSA and differential activity against *E. coli* and *A. baumanii*. Only one compound, **26**, showed activity against *K. pneumoniae* (MIC*_E. coli_* = 9.3 µM, MIC*_K. pneumoniae_* = 9.3 µM, MIC*_A. baumanii_* = 4.7 µM, MIC*_MRSA_* = 1.2 µM, Figure 9) and no activity was reported against *P. aeruginosa*. Three complexes showed synergistic activity when co-administered with the antibiotic vancomycin against vancomycin resistant Enterococci. Moreover, a subset of the compounds also exhibited good activity against the fungal strain *C. albicans* and *C. neoformans*. Some compounds could also disrupt *S. aureus* biofilm formation [103]. Comparison of the structures of the 14 complexes reveal that the biphenyl-Cp ligand is essential, as the pentamethyl-Cp or the mono-phenyl-Cp showed greatly diminished antibacterial activity. Similarly, the biguanine ligand on its own was also not active, indicating that the overall structure of the complex plus the iridium centre are required for the observed biological action. The authors found that the antibacterial activity of the complexes was very similar under aerobic and anaerobic conditions against *S. aureus* and *S. pyogenes*, indicating that ROS generation is not required for their antimicrobial effect. Further essays showed that bacterial cell envelopes remained intact upon treatment with the iridium compounds. Finally the authors hypothesize that Ir(III) biguanine complexes could enter bacteria and then undergo ligand exchange reactions with thiol-containing biomolecules, releasing the biguanide ligands, which might interfere with cell processes inside the bacteria [103].

Iridium complexes seem to have good antimicrobial potential, however, the cost of the element is similar to the cost of gold [104]. As antibiotics are generally taken in quite high doses and are not priced as highly as e.g., anti-cancer treatments, iridium-antibiotics are unlikely to be commercially viable. Nevertheless, as a last resort, a super-effective iridium antibiotic would be better than no option at all.

## 8. Rhenium

**Figure 10:**
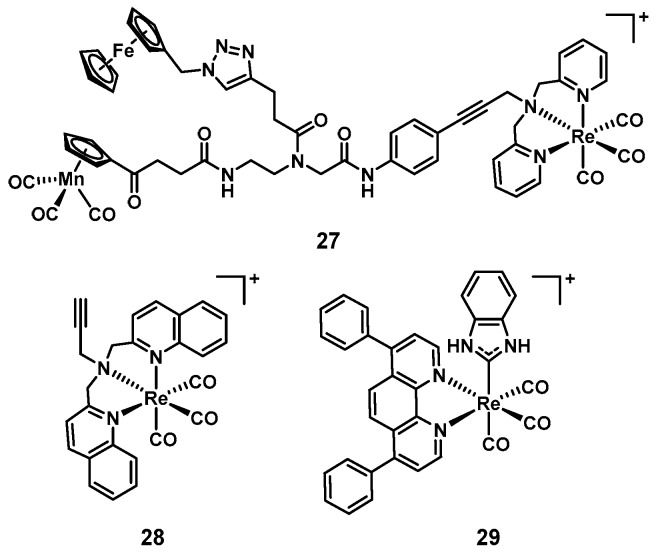
Structures of rhenium complexes with reported antimicrobial activity.

Rhenium complexes have slowly but steadily gained attention for their medicinal applications. A couple of reviews on their anticancer properties have recently been published [105,106]. In line with what is generally true for the field, their antimicrobial activities have only been studied sparsely. The groups of Metzler-Nolte and Bandow reported a series of studies into the structure–activity relationship of a group of trimetallic complexes. Amongst these compounds, **27** showed excellent activity against Gram(+) bacteria, including MRSA (Figure 10). The authors also found that the [(dpa)Re(CO)_3_] moiety was crucial for the observed activity [107,108]. Further experiments revealed that this class of compounds disturbed processes at the bacterial cell membrane such as respiration and cell wall biosynthesis [109]. Inspired by these results, we recently investigated bisquinoline rhenium tricarbonyl complexes for their antibacterial properties. The most promising compound (**28**, Figure 10), showed good activity against MSSA and MRSA (MIC = 4–8 µg/mL), but was inactive against Gram(−) bacteria. Upon light irradiation at 365 nm, the activity of the compound increased significantly, the effect also imparting Gram(−) activity against wildtype and colistin-resistant *E. coli* (MIC = 4–8 µg/mL). The compound showed no haemolytic properties and a 20-fold lower toxicity against human HEK cells compared to its MIC against *S. aureus*. The distinct activity profile in the absence and presence of light indicates a dual-mode of action [110]. In 2017 Siegmund et al., described the preparation of novel Re(I) *N-*heterocyclic carbene (NHC) complexes. These compounds were found to possess potent antibacterial activity against Gram(+) strains, while being inactive against Gram(−) ones (**29** shown as example, MIC*_B. subtilis_* = 0.7–1.3 µM, MIC*_S. aureus_* = 0.7 µM, Figure 10) [111]. This new class of rhenium complexes has also been patented by the groups and is currently being developed further [112].

While still in its infancy, rhenium has proven itself a promising starting point for the development of new classes of antibacterial agents, in particular against Gram(+) strains. Again, there is a still total lack of in vivo data for this class of compounds which will have to be addressed in future studies.

## 9. Metal Complexes vs. Organic Molecules

It is evident that the tip of the iceberg has only barely been scratched when it comes to investigating the antimicrobial potential of metal complexes (Table 2).

Although there have been in vitro studies into many other elements, including chromium [115], iron [116], manganese [117,118,119,120], copper [121], rhodium [122], palladium [123,124], and platinum [125], these were often preliminary in nature and require further validation [27,124,126]. While some metal complexes and metal ions have been shown to possess excellent antimicrobial activity, the question remains whether or not metals offer any significant advantages over purely organic compounds. A common misconception amongst non-inorganic chemists is the notion that metals and their complexes are just generally toxic. To start to address this issue, we analyzed the antimicrobial profile of close to 1000 metal complexes [127]. These compounds were screened by the Community for Open Antimicrobial Drug Discovery (CO-ADD), a global antimicrobial screening platform that screens user-submitted compounds against critical ESKAPE pathogens and two fungal species for free [128,129,130]. Since 2015, the CO-ADD has profiled almost 300,000 compounds under systematic and reproducible conditions. The 906 evaluated metal complexes displayed a hit-rate of 27% against the tested panel (a hit being defined as having at least one MIC ≤ 16 µg/mL or 10 µM). This contrasts with the substantially lower hit-rate found for purely organic molecules in the CO-ADD database (1.6%). Strikingly, this difference was not attributable to increased metal toxicity, as the toxicity rates for both compound classes, as measured by cytotoxicity against HEK293 cells and hemolytic effects against human red blood cells, was found to be similar (64.5% vs. 64.2%). Removal of cytotoxic and/or hemolytic compounds still left an overall hit-rate of 9.9% for metal compounds, compared to 0.87% for the rest of the CO-ADD database [127]. While this dataset is still rather small, this initial analysis further underscores the potential that metal complexes could bring to the antibiotic drug discovery pipeline.

When it comes to their development as potential drugs, organic molecules do have some advantages compared to metal complexes. Firstly, there is an immense amount of knowledge that has been accumulated over the years on the pharmacological and metabolic behavior of organic compounds. For metal complexes this is still uncharted territory that will require many years of costly and lengthy experiments to explore. The toolkit for the synthesis of organic molecules is vast, with almost any conceivable transformation possible and, more importantly scalable for industrial production. The knowledge of such processes with metal complexes is also rather limited. As stated before, the cost of the metal can be detrimental to the drug development process, particularly in the field of antibiotics, where dosages are high, market competition is harsh, and margins are small. Nevertheless, with costly metals, the compounds could still be developed as last resort-type antibiotics. In this case, the high price could have a beneficial effect as it would discourage indiscriminate use by patients. On the other hand, compounds based on more affordable transition metals do not suffer from this drawback.

As is highlighted in this article, there is clearly much untapped potential in metal complexes for antimicrobial applications. This is evidenced by the increased activity in the field of metal-based antibiotics over the last decade. Despite the head start that organic molecules have in the drug development world, metals do bring some unique advantages to the table.

Metal complexes have access to multiple unique modes of action. They can undergo ligand exchange reactions, release bioactive molecules or be triggered by light irradiation to generate ROS. Alessio and coworkers have previously classified the possible modes of action for anticancer metal compounds and these possibilities are certainly valid for antibiotic applications as well [131]. Some reports have already described the application of metal compounds as catalytic metallodrugs against cancer where the metal complex generates a bioactive compound in situ (or depletes the cells of essential substrates) [132,133,134,135]. On top of this, coordination compounds have access to a wide range of 3D geometries. Three-dimensionality of compounds has been associated with higher clinical success in previous work [3,4]. Indeed, a recent study found that metallofragments show excellent potential for fragment-based drug discovery approaches as they cover a higher degree of available three-dimensional chemical space [8].

While some studies, such as the work of Goss et al., found resistance rates comparable to conventional antibiotics with metal complexes, other reports, such as those by Dwyer [69], Sun [67], Fuchs [47], or Sadler [103] found no resistance development even after many rounds of treatment. Since the ROS generated in aPDT treatments has many possible targets, it is difficult for bacteria to develop resistance mechanisms against this therapy. Indeed, no conclusive evidence of resistance against aPDT has been reported to date [75,77]. In general, further work is required to probe the capability of bacteria to develop resistance against metal complex-based treatments. But so far the data suggest that metal-compounds are less likely to induce resistance in bacteria.

Another aspect that needs to be included in all future studies of metal complexes are detailed experiments into their stability in water, in the presence of biological media, and human blood. While the synthesized compounds do not necessarily have to be the active drug in the patient (such as is the case for cisplatin), it is imperative that we gather more systematic knowledge about the stability and solution behavior of these compounds. Finally, the recent tour de force by the group of Sun illustrates how cutting-edge technology can be used to elucidate the detailed mechanisms of action of metal compounds [136].

Generally, only very limited in vivo efficacy data are available for metal complexes, hindering the further development of potentially promising compounds at this stage. Hopefully future studies will follow the preclinical development path laid out by medicinal organic chemists over the last decades and lead to a better understanding of the in vivo behavior of metal complexes.

It is clear that the golden age of metalloantibiotics is still ahead of us. The number of tested metal-containing compounds is dwarfed by the millions of organic compounds that have been studied to date, highlighting an information gap ripe for filling by the inorganic chemistry community. At the same time, many metals remain unexplored, with the promise of low-hanging fruit for researchers everywhere. There are almost no literature reports for the metals cobalt, nickel, molybdenum, tungsten, and osmium, making these elements interesting starting points for new investigations.

Taken together, the future is bright for this field and the coming decade will likely see many more promising studies on metal-based antibiotics. Optimistically, it is anticipated that a metal-based antibiotic drug candidate will reach clinical trials within the next 10 years. 

## Figures and Tables

**Table 1:** **Minimum inhibitory concentration** (MIC) values for auranofin analogues against a series of Gram(+) and Gram(−) strains (in µM).

	*Ab* ^a^	*Pa* ^b^	*Ec* ^c^	*Kp* ^d^	*Sa* ^e^	*Ef* ^f^	*Ec* ^g^	*Hp* ^h^
**Aur ^i^**	47	377	189	377	0.04	0.09–0.2	24	n.d.
**1**	4–17	>547	4–9	34	0.3–0.5	0.3–0.5	9	n.d.
**2**	9–17	>547	4	34	0.3	0.3–0.5	9	n.d.
**3**	3–6	23–91	3	11	0.3	0.3	1–6	n.d.
**4**	8–16	>503	8–16	31	0.5	0.2–0.5	8–31	n.d.
**5**	24	189	47	189	0.04–0.09	0.09	12	0.3
**6**	15–29	464	116	464	0.02	0.05–0.1	4–7	0.35

^a^*Acinobacter baumannii* NCTC 13420, ^b^ *Pseudomonas aeruginosa* NCTC 13437, ^c^ *Enterobacter cloacae* NCTC 13405, ^d^ *Klebsiella pneumoniae* ATCC 700603, ^e^ *Staphylococcus aureus* JE2 (USA300), ^f^ *Enterococcus faecium* NATCC 700221, ^g^ *Escherichia coli* NATCC 25922, ^h^ *Helicobacter pylori* G27, ^i^ Auranofin.

**Table 2:** Overview of metal complexes discussed in this article.

Compound	# ^a^	Metal	G(+) ^b^	G(−) ^c^	Media ^d^	Target/MoA ^e^	Cytotoxicity ^f^	*In vivo* ^g^
**AgNO_3_** [113]	1	Ag	Yes	Yes	LB	TCA cycle	Yes (Yes)	Yes (human)
**Aur **[15]	1	Au	Yes	No	CAMBH	Trx inhibition	Yes (Yes)	Yes (mouse)
**1–6** [16]	6	Au	Yes	Yes	CAMBH	Trx inhibition	Yes (some)	n.d.
**Ganite **[50]	1	Ga	No	Yes	MH; HS	Fe-metabolism [61]	Yes (No)	Yes (human)
**Ga(DFO)** [53]	1	Ga	n.d.	Yes	TSB	Fe-metabolism [61]	Yes (No)	Yes (rabbit)
**Ga(PPIX)** [51]	1	Ga	Yes	Yes	LB, MHB, DMHB, RPMI-HS	Fe-metabolism [61] Cytochrome [114]	Yes (No)	Yes (sheep)
**CBS **[64]	1	Bi	Yes	Yes	TSB	Multiple targets and MBLs [64]	Yes (No)	Yes
**7 **[70]	10	Ru	Yes	n.d.	DFH	n.d.	Yes (Yes)	Yes (mouse)
**8 **[73]	3	Ru	Yes	No	LB, BHI	DNA intercalation	n.d.	Yes (fungi)
**9** [78]	3	Ru	Yes	Yes	MH	PDT	n.d.	n.d.
**10 **[79]	1	Ru	Yes	No	MH	PDT	Yes (No)	n.d.
**11 **[79]	1	Ru	Yes	Yes	MH	PDT	Yes (No)	n.d.
**12 **[80]	17	Ru	Yes	Yes	LB	PDT	Yes (Some)	n.d.
**13 **[81]	3	Ru	Yes	Yes	LB	PDT	Yes (No)	n.d.
**14 [82]**	1	Ru	No	No	TSB	Light-triggered isoniazid release	Yes (No)	n.d
**15 **[83]	2	Ru	Yes	Yes	CAMBH	β-lactamase	n.d.	n.d.
**16 **[85]	26	Ru	Yes	Yes	CAMBH	RNA, ribosome	Yes (Some)	n.d.
**17–18** [89]	14	Ru	Yes	Yes	CAMBH	Bacterial membrane	Yes (Some)	n.d.
**19 **[91]	3	Ru	Yes	Yes	CAMBH	Bacterial membrane	Yes (Some)	n.d.
**20** [92]	4	Ru	Yes	Yes	CAMBH CDM	Bacterial membrane, DNA	Yes (No)	Yes (moth)
**21** [96]	5	Ir	Yes	No	LB	Not studied	Yes (Yes)	n.d.
**22** [97]	6	Ir	Yes	Yes	MH	Binds DNA	n.d.	n.d.
**23** [99]	3	Ir	n.d.	Yes	LB	Not studied	n.d.	n.d.
**24 **[101]	16	Ir	Yes	n.d.	MH	Not studied	Yes (No)	n.d.
**25 **[102]	8	Ir	Yes	n.d.	MH	Not studied	Yes (No)	Yes (mouse)
**26 **[103]	14	Ir	Yes	Yes	CAMBH	Biguanine ligand release	Yes (Some)	n.d.
**27 **[108]	13	Re	Yes	No	MH	Cell wall synthesis, respiration	Yes (Some)	n.d.
**28 **[110]	3	Re	Yes	Yes	CAMBH	PDT	Yes (Some)	n.d.
**29 **[111]	10	Re	Yes	No	MH	Not studied	n.d.	n.d.

^a^ Number of compounds reported in the cited study; ^b^ Whether the compounds were active against any Gram(+) strains; ^c^ Whether the compounds were active against any Gram(−) strains; ^d^ growth media used for antibacterial activity determination; ^e^ Putative target and or mode of action (MoA) for the compound(s); ^f^ Whether cytotoxicity against human cells was determined (if it was found to be cytotoxic). Note: For this article a compound was considered cytotoxic if the CC50 or IC50 was <50 µM. It is noted that most compounds were tested against different cell lines and the reader is referred to the cited publications for further details. ^g^ Whether the compounds were evaluated in vivo (animal model). n.d.: not determined LB: Lysogeny broth; CAMBH: cation-adjusted Mueller–Hinton broth; MH: Mueller-Hinton broth; HS: human serum; TSP: tryptic soy broth; DFH: Difco heart-infusion broth + 10% horse serum; BHI: Brain Heart Infusion broth; CDM: Chemically defined medium; MBL: metallo-β-lactamases; PDT: photodynamic therapy.
